# A Platoon-Based Adaptive Signal Control Method with Connected Vehicle Technology

**DOI:** 10.1155/2020/2764576

**Published:** 2020-06-01

**Authors:** Ning Li, Shukai Chen, Jianjun Zhu, Daniel Jian Sun

**Affiliations:** ^1^Ulanqab Vocational College, Ulanqab 012000, Inner Mongolia, China; ^2^State Key Laboratory of Ocean Engineering, School of Naval Architecture Ocean and Civil Engineering, Shanghai Jiao Tong University, Shanghai 200240, China; ^3^Smart City and Intelligent Transportation (SCIT) Center, Shanghai Jiao Tong University, Shanghai 200240, China; ^4^Institute of Computing Technologies, China Academy of Railway Sciences Corporation Limited, Beijing 100081, China

## Abstract

One important objective of urban traffic signal control is to reduce individual delay and improve safety for travelers in both private car and public bus transit. To achieve signal control optimization from the perspective of all users, this paper proposes a platoon-based adaptive signal control (PASC) strategy to provide multimodal signal control based on the online connected vehicle (CV) information. By introducing unified phase precedence constraints, PASC strategy is not restricted by fixed cycle length and offsets. A mixed-integer linear programming (MILP) model is proposed to optimize signal timings in a real-time manner, with platoon arrival and discharge dynamics at stop line modeled as constraints. Based on the individual passenger occupancy, the objective function aims at minimizing total personal delay for both buses and automobiles. With the communication between signals, PASC achieves to provide implicit coordination for the signalized arterials. Simulation results by VISSIM microsimulation indicate that PASC model successfully reduces around 40% bus passenger delay and 10% automobile delay, respectively, compared with signal timings optimized by SYNCHRO. Results from sensitivity analysis demonstrate that the model performance is not sensitive to the number fluctuation of bus passengers, and the requested CV penetration rate range is around 20% for the implementation.

## 1. Introduction

Signal light plays a significant role in urban traffic management and control. Adaptive signal control, a state-of-the-art type of traffic control, can remarkably improve the mobility around signalized intersections compared with fixed or actuated controls [1, 2]. In urban areas, the number of transit buses has boosted in recent years. With proper control strategy favoring the bus and high-occupied vehicles, the controller can effectively reduce passenger delay at intersections. However, recent studies found that the transit signal priority control strategies (TSP) inevitably interrupt automobile traffic flow and increase the control delay for automobile users [3]. To minimize individual traveler delay at intersections, it is necessary to develop a balanced multimodal signal control strategy.

One significant challenge in multimodal adaptive signal control lies in the short-time traffic prediction for cars and transit buses. With the advances in connected vehicle (CV) technology [4], communication between vehicle-infrastructure (V-I) enables reliable traffic information collection. Based on accurate vehicle location and speed data, the generated signal timings may readily match traffic demand fluctuation. Therefore, the CV technology should be incorporated, thus enhancing the multimodal control reliability in traffic management, particularly for signalized intersections.

Diakaki et al. [3] conducted a comprehensive review on recent multimodal control strategies. Some typical adaptive signal control systems, such as SCOOT [5] and SCATS [6], apply a rule-based strategy to grant signal priority to transit buses. Upon receiving a priority request, the controller's reactions involve green extension, red truncation, and special stages. For example, Skabardonis and Geroliminis [7] proposed a strategy to reduce bus control delays and minimize adverse effects on the rest of the traffic. In addition, Wadjas and Furth's control strategy [8] aims to assign priority to light rail transits in 3-4 cycles ahead, so that the dwell time became an important factor in arrival estimation. Although the rule-based control strategy is a proactive approach to adjust signal timing for reducing bus delay, it generally deals with only one priority request during the given period. Thus, its application is restricted under oversaturated demand with high bus frequency.

Optimization-based strategies attempt to minimize total disutility (delay, queue length, and stop numbers) by employing nonlinear [9], mix-integer linear [10], or dynamic programming [11]. One advantage of such a control strategy is the nonlimitation on the number of conflicting priorities in the optimization model, compared with rule-based strategies. Christofa et al. [9] developed a model to minimize the total personal delay in mixed traffic lanes based on assumptions that automobile arrival follows a uniform distribution, which is not suitable for closely located intersections. To reduce the potential bus delay at downstream, Ramezani et al. [12] introduced a TSP strategy that comprises of different models to estimate car and bus delays, respectively, for the one-way arterials. However, both strategies assumed an exclusive lane for transit vehicles, indicating that the queues in front of the bus were largely neglected.

To address the disadvantages of the conventional strategies in modelling the mixed traffic conditions, recent research efforts have been dedicated to developing new control methods based on the CV data. He et al. [13] proposed a novel control model called PAMSCOD, which clusters vehicles into platoons to incorporate the arrival patterns subject to upstream intersections. A mixed-integer programming was introduced to solve the problems online based on the platoon or bus serving requests. Hu et al. [14] further exploited the CV technology by adjusting bus speed to ensure the “green wave” under a pair of intersections. Furthermore, Zeng et al. [15] proposed a control strategy named PAPSCCI to model the dynamics of vehicular arrivals. Although vehicles were treated individually to improve the performance, the computational time may be significantly increased under saturated demands.

One common characteristic of the control strategies is the centralized control, which aggregates all signals within one region into a global optimization. Consequently, the increasing number of intersections and the complexity in traffic dynamics often render the problem nonscalable [16]. Therefore, how to achieve progression for multiple traffic modes under a decentralized framework is still a challenging research problem [17].

This study proposes a platoon-based adaptive signal control (PASC) strategy based on the vehicular information extracted from CV technology. A mixed-integer linear programming (MILP) is developed for online optimization [18]. The main difference between PAMSCOD and the proposed model lies in platoon arrival and delay formulation. In the proposed model, the residual platoon is considered by the constraint so that the delay approximation is simplified. Moreover, the mechanism about implicit coordination is introduced to achieve the progression of neighboring intersections. Under the framework of decentralized control, PASC can save computation time and thus has the potential to be applied in a large urban network. The remainder of the paper is organized as follows. The details of the PASC strategy, as well as the mechanism of implicit coordination between controllers, are proposed in Section 2. In Section 3, a simulation and evaluation platform is developed based on VISSIM. Then, an arterial road, Dongchuan Road, in Minhang District, Shanghai, was modeled to demonstrate model effectiveness. Finally, Section 5 summarizes the conclusions and offers the suggestions for further studies.

## 2. Platoon-Based Adaptive Signal Control

### 2.1. Platoon Identification

The critical headway is used to identify the approaching and stopping platoons at intersections. Suppose that *h*_0_ is the critical headway under 100% CV penetration rate, then under the penetration rate of *R*_*p*_, the critical headway is denoted as *h*_*p*_ and calculated as in equation (1). If the detected headway of two consecutive connected vehicles is less than *h*_*p*_, the vehicles are treated as part of a platoon; otherwise, they are regarded as individual ones:

(1) $$\begin{array}{c} h_{p}=\frac{h_{0}}{R_{p}}. \end{array}$$

At the same time, with penetration rate *R*_*p*_, the number of vehicles in the platoon is *n*_*p*_, as calculated in the following equation:

(2) $$\begin{array}{c} n_{p}=\frac{N}{R_{p}}, \end{array}$$

where *h*_0_ indicates the critical time headway which is recognized as platoon and *N* is the detected number of connected vehicles within a platoon. Moreover, the platoon arrival time at stop line is estimated based on the velocity and position of the leading vehicle.

### 2.2. Mixed-Integer Linear Programming Model in PASC

This section introduces a mixed-integer linear programming (MILP) model for multimodal traffic control optimization. The objective is to minimize the total delay for automobile and bus passengers. The set of constraints incorporates NEMA configuration and traffic dynamics upon the stop bar, including serving cycle estimation, platoon arrival, and split. Variables and data notations for the optimization model are listed in Table 1).

#### 2.2.1. Phase Precedence Constraints

**Figure 1:**
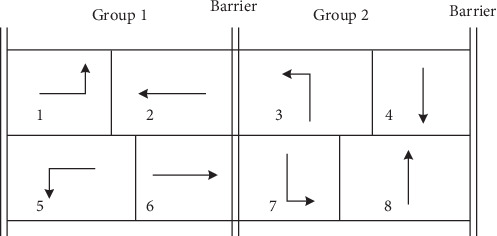
NEMA phase with dual-ring structure.

The proposed model is based on the standard NEMA 8-phase configuration.  Figure 1 presents the phase structure and precedence constraint for a four-leg intersection [10, 19]. Each ring contains four movements, in which conflicting movements are separated by a barrier so that the first movement in one phase group starts only after all movements in the previous group terminate. The phase precedence constraints are incorporated into the optimization model for each intersection in the control zone.

In this study, the phase precedence constraints (3)–(12) are introduced so that MILP can be formulated at any phase pair. Under NEMA framework, currently active phases belong to following eight phase combinations: 1 and 5, 1 and 6, 2 and 5, 2 and 6, 3 and 7, 3 and 8, 4 and 7, and 4 and 8. Thus, NEMA origin phase pair is crucial to calculate the elapsed green time and the minimum green time constraint for current phases. To simplify the model, each intersection is assumed to have the same phase sequence as presented in Figure 1, in which phase pair 1 and 5 is set as the origin phase of a cycle. Then, similar to PAMSCOD [13], the phase precedence constraint is listed as follows:

(3) $$\begin{array}{c} t_{1,1}=O_{1}+Y+R\;t_{5,1}=O_{5}+Y+R, \end{array}$$

(4) $$\begin{array}{c} t_{2,k}=t_{1,k}+v_{1,k}+s_{1,k},\\ t_{4,k}=t_{3,k}+v_{3,k}+s_{3,k},\\ \forall k, \end{array}$$

(5) $$\begin{array}{c} t_{6,k}=t_{5,k}+v_{5,k}+s_{5,k},\\ t_{7,k}=t_{6,k}+v_{6,k}+s_{6,k},\\ t_{8,k}=t_{7,k}+v_{7,k}+s_{7,k},\\ \forall k, \end{array}$$

(6) $$\begin{array}{c} t_{1,k+1}=t_{4,k}+v_{4,k}+s_{4,k},\\ t_{5,k+1}=t_{8,k}+v_{8,k}+s_{8,k},\\ \forall k, \end{array}$$

(7) $$\begin{array}{c} g_{1,k}+g_{2,k}=g_{5,k}+g_{6,k},\\ g_{3,k}+g_{4,k}=g_{7,k}+g_{8,k},\\ \forall k, \end{array}$$

(8) $$\begin{array}{c} v_{p,k}=g_{p,k}+Y+R,\;\forall p,k, \end{array}$$

(9) $$\begin{array}{c} g_{p,1}=g_{p}^{N},\;\forall p\in \Delta_{p}, \end{array}$$

(10) $$\begin{array}{c} s_{p,k}=0\;\forall p,k\geq 2\text{ or }p\notin \Delta_{1}\cup \Delta_{2},k=1, \end{array}$$

(11) $$\begin{array}{c} G_{p}^{\min}\leq g_{p,k}\leq G_{p}^{\max},\;\forall p,k\geq 2\text{ or }p\notin \Delta_{p},k=1, \end{array}$$

(12) $$\begin{array}{c} g_{p,k}\geq \max\left\{E_{p},G_{p}^{\min}\right\},\;\forall p\in \Delta_{1}\cup \Delta_{2},\;k=1. \end{array}$$

Constraints (3)–(6) represent the phase sequence for dual-ring phase structure, and the barrier constraint between two phase groups is modeled by constraint (7). It is assumed that each phase starts with clearance time, and consequently, constraint (3) indicates the initial green start time within a cycle. A phase set Δ_*p*_ represents the past phases, in which the green time duration is determined by constraint (9). For currently active phases denoted in sets Δ_1_ and Δ_2_, the minimum green time constraint is defined as the larger value of the elapsed green time and the minimum green time as shown in constraint (12). Considering that extra time is possible to be required at current phases to satisfy the barrier constraint [13], the slack variable *s*_*p*,*k*_ is defined to relax the maximum green time constraint as constraint (10).

**Figure 2:**
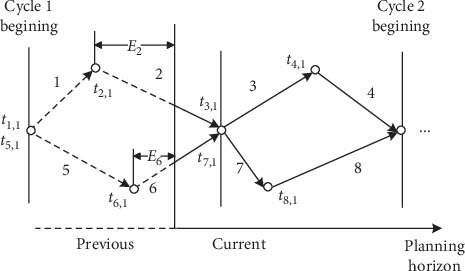
Example of planning horizon and current phases.

Figure 2 presents the case that the precedence constraint is formulated. The current horizon starts from phases 2 and 6 with elapsed green time E2 and E6, respectively. The dotted line means the green time for past phases, while the solid line represents the green time to be determined. In this study, the planning horizon is assumed to contain at least two complete cycles after the current cycle. Under this phase precedence constraint, the cycle length is flexible to traffic demand fluctuation.

#### 2.2.2. Traffic Dynamics and Delay Evaluation

In the previous research, delay was used as the most common performance index to evaluate the quality of signal timings [9, 15, 20]. This paper classifies the platoon delay into two types: (1) *D*_*p*,*j*,*m*_^*l*^: total delay by stopping the leading vehicle of platoon (*p*, *j*, *m*), including the time that leading vehicle waits for the end of red time and the departure of front platoons; (2) *D*_*p*,*j*,*m*_^*s*^: total delay by splitting platoon (*p*, *j*, *m*) if the green duration is not enough to discharge the entire platoon. Before delay evaluation, it is necessary to estimate the cycle that the platoon can cross the intersection.

**Figure 3:**
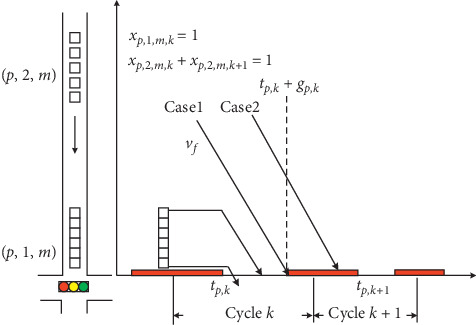
Platoon serving cycle estimation.

The serving cycle estimation is based on the platoon arrival time at the stop line and the possible delay by stopping the leading vehicle of platoon.  Figure 3 illustrates the two possible cycles serving the platoons around the intersection. For platoon (*p*, 1, *m*), it is clear that it will pass the stop line in cycle *k*. Meanwhile, whether the leading vehicle of platoon (*p*, 2, *m*) can pass the intersection at cycle *k* depends on the free-flow speed and the amount of time waiting for the discharge of platoon (*p*, 1, *m*). The traffic dynamics are described by the following constraints:

(13) $$\begin{array}{c} R_{p,j,m}+D_{p,j,m}^{l}-\left(t_{p,k}+g_{p,k}\right)\leq \left(1-x_{p,j,m,k}\right)M,\;\forall \left(p,j,m\right)\in \Gamma ,k, \end{array}$$

(14) $$\begin{array}{c} \sum_{k}x_{j,p,m,k}=1,\;\forall \left(p,j,m\right)\in \Gamma , \end{array}$$

where *R*_*p*,*j*,*m*_+*D*_*p*,*j*,*m*_^*l*^is the actual platoon departure time considering the delay by stopping the leading vehicle of platoon, *t*_*p*,*k*_+*g*_*p*,*k*_ is the end of green time for phase *p* in cycle *k*, and *x*_*p*,*j*,*m*,*k*_ indicates that whether platoon (*p*, *j*, *m*) departs during cycle *k*.

As depicted in Figure 3, it is figured out that if the leading vehicle of platoon (*p*, 2, *m*) arrives at stop line before the end of green time (Case 1), then cycle *k* is selected as serving cycle. Otherwise, the entire platoon has to wait until the beginning of green time in cycle *k* + 1 (Case 2). Constraint (14) ensures that only one cycle in the planning horizon is selected to serve the leading vehicle of the platoon.

**Figure 4:**
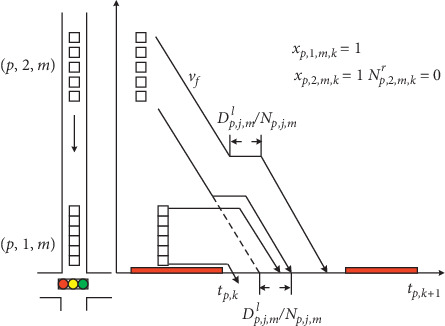
Delay incurred by stopping the leading vehicle in platoon (*j* = 2).

For simplicity, the platoon is assumed to travel through the intersection with no dispersion effect. Consequently, all vehicles within one platoon keep the same headway and the vehicle trajectories are parallel all time based on kinematic theory [21]. This means that all vehicles within the platoon experience the same delay, as shown in Figure 4. Therefore, the average delay is denoted as (*D*_*p*,*j*,*m*_^*l*^/*N*_*p*,*j*,*m*_), which is the difference between the possibly earliest departure time of the leading vehicle and the estimated arrival time at stop line with free-flow speed. To this end, the delay by stopping the leading vehicle in platoon is presented as follows:

(15) $$\begin{array}{c} \;\left(\frac{D_{j,p,m}^{l}}{N_{p,j,m}}\right)\geq t_{p,k}+\sum_{m}\sum_{j_{1}=1}^{j_{1}<j}N_{p,j_{1},m}\cdot \frac{h_{p}}{l_{p}-R_{p,j,m}-\left(1-x_{p,j,m,k}\right)M},\;\forall p,k=1\text{ or }p\in \Delta_{p},k=2,\;\left(p,j,m\right)\in \Gamma , \end{array}$$

(16) $$\begin{array}{c} \left(\frac{D_{j,p,m}^{1}}{N_{p,j,m}}\right)\geq t_{p,k}+\sum_{m}\sum_{j_{1}=1}^{j_{1}<j}N_{p,j_{1},m}^{r}\cdot \frac{h_{p}}{l_{p}-R_{p,j,m}-\left(1-x_{p,j,m,k}\right)M},\;\forall p,k>2\text{ or }p\notin \Delta_{0},k=2,\;\left(p,j,m\right)\in \Gamma , \end{array}$$

where  *t*_*p*,*k*_+∑_*m*_∑_*j*_1_=1_^*j*_1_<*j*^*N*_*p*,*j*_1,*m*__ · *h*_*p*_/*l*_*p*_ represents the earliest departure time for the leading vehicle of platoon (*p*, *j*, *m*) in the first cycle length from the moment the MILP is formed, including the phases after Δ_1_ and Δ_2_ in current cycle and next cycle's phases belonging to Δ_0_. For subsequent phases, the residual part of preceding platoons *N*_*p*,*j*_1_,*m*_^*r*^ should be considered in constraint (16). The earliest departure time is the summation of starting time of phase *p* and the amount of time for discharging platoons in front of  (*p*, *j*, *m*). The effects of the multiple lanes to saturated headway are considered by introducing the lane number *l*_*p*_.

Figure 4 illustrates the calculation for the delay by stopping the leading vehicle delay *D*_*p*,*j*,*a*_^*l*^. Platoon (*p*, 2, *m*) is traveling towards the intersection while platoon (*p*, 1, *m*) has already been stopped at the stop line. Because platoon (*p*, 1, *m*) needs *N*_*p*,1,*m*_ · (*h*_*p*_/*l*_*p*_) duration for discharging, platoon (*p*, 2, *m*) may consequently join the platoon tail and thus experience delays. It should be noted that since the green duration is enough for discharging all vehicles in platoon  (*p*, 2, *m*), platoon split does not occur and no vehicles are left behind.

**Figure 5:**
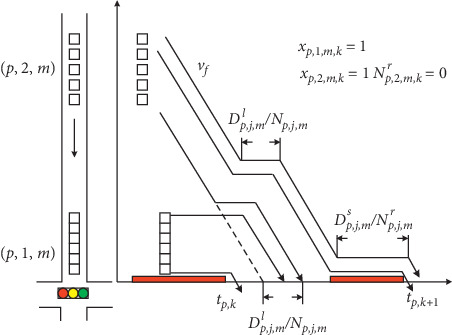
Delay incurred by splitting platoon (*j* = 2).

If the assigned green time is not enough to serve all vehicles within a platoon, the platoon will be split into two components, and the remaining part has to experience extra control delay *D*_*p*,*j*,*a*_^*s*^, i.e., red duration between cycle *k* and cycle *k* + 1. Based on the assumption with no dispersion, it is identified that each vehicle within the residual platoon experiences the same delay duration as depicted in Figure 5.

Figure 5 illustrates the generation of delay incurred by splitting the moving platoon. First, the platoon (*p*, 2, *m*) is stopped to wait for discharge of the front queues. After the platoon restarts, only a part of vehicles departures from the stop line, while the remaining vehicles become the first platoon for cycle *k* + 1. Since the bus is treated as a special platoon with individual vehicle, the size of residual platoon is not considered. The number of residual platoon before the end of cycle *k* is then formulated as follows:

(17) $$\begin{array}{c} N_{p,j,m,k}^{r}\geq N_{p,j,m}-\frac{t_{p,k}+g_{p,k}-R_{p,j,m}-D_{p,j,m}^{l}}{h_{p}\cdot l_{p}}-\left(1-x_{p,j,m,k}\right)M,\;\forall \left(p,j,m\right)\in \Gamma ,k,m=a, \end{array}$$

where  *t*_*p*,*k*_+*g*_*p*,*k*_ − *R*_*p*,*j*,*k*_ − *D*_*p*,*j*,*m*_^*l*^  represents the remaining green time for discharging platoon (*p*, *j*, *m*) when the leading vehicle in the platoon can pass through intersection during cycle *k*. Two situations may occur if the number of remaining vehicles *N*_*p*,*j*,*m*,*k*_^*r*^  is equal to 0. The first is that the leading vehicle of platoon  (*p*, *j*, *m*)  does not arrive at the stop line in cycle *k*, and the second is that the assigned green time allows the entire platoon to depart from the intersection. Both scenarios do not produce the platoon splitting delay.

Given the number of residual vehicles in platoon (*p*, *j*, *m*)  before the end of cycle *k*, the total delay incurred by splitting platoon can be presented as follows:

(18) $$\begin{array}{c} D_{j,p,m}^{s}\geq N_{j,p,m,k}^{r}\cdot \left(t_{p,k+1}-t_{p,k}-g_{p,k}\right),\;\forall \left(p,j,m\right)\in \Gamma ,k, \end{array}$$

where *t*_*p*,*k*+1_ − *g*_*p*,*k*_ − *t*_*p*,*k*_ is the effective red duration between cycle *k* and *k *+ 1. Unfortunately, this expression makes the constraint nonlinear. Consequently, the delay incurred by splitting platoon is approximated by replacing the component of effective red time with nominal effective red time [20, 22]. Thus, constraint (18) can be reformulated as follows:

(19) $$\begin{array}{c} D_{p,j,m}^{s}\geq N_{p,j,m,k}^{r}\cdot \left(C^{N}-g_{p}^{N}\right),\;\forall \left(p,j,m\right)\in \Gamma ,k, \end{array}$$

where *C*^*N*^ represents the nominal cycle length.

#### 2.2.3. Model Formulation

In the proposed model, the objective function is to minimize the total passenger delay for automobile and bus users, which is achieved by weighting delay by vehicular passenger occupancy collected by CV technology. Compared with the vehicle-based delay objective function, the person-based delay objective function can better address conflicting priority for bus services [23]. A summary of model is presented as follows:

(20) $$\begin{array}{c} \begin{array}{cc} \text{objective function}: & \sum_{\left(j,p,m\right)\in \Gamma }{\text{Occ}}_{p,j,m}\cdot \left(D_{p,j,m}^{l}+D_{p,j,m}^{s}\right)\\ \text{s.t.} & \begin{array}{c} \text{phase precedence constraint}:\left(3\right)-\left(12\right)\\ \text{serving cycle estimation:}\left(13\right)\text{and}\left(14\right)\\ \text{delay by stopping leading vehicle}:\left(15\right)\text{and}\left(16\right)\\ \text{number of residual queue:}\left(17\right)\\ \text{delay by splitting platoon}:\left(19\right), \end{array} \end{array} \end{array}$$

where Occ_*p*,*j*,*m*_ is the passenger occupancy for platoon (*p*, *j*, *m*), and the controlled variables are binary decision variables, and all other variables are nonnegative.

As discussed, the proposed control model aims to serve all identified platoons in three cycles under the framework of rolling horizon. The optimization model is formed and solved every 30 s to implement the signal control. For simplicity, only two significant platoons were considered for noncoordinated phases, while at least one platoon at upstream intersection is estimated for the coordinated phases (phases 2 and 6). Since the queues at upstream stop bar will approach to the current intersection as one or more platoons, the size and starting time of those platoons could be roughly estimated by the location of upstream vehicles and the signal timing plans [13]. Location data on the upstream vehicle are collected by CV technology, while the signal timing is extracted through communication between signals.

### 2.3. Communication between Signals

For a signalized arterial, the proposed control model optimizes signal timing for each intersection in a fixed sequence. The implicit coordination depends on optimized signal timings of individual controller and the way of unknown signal timing estimation. In this study, the latest historical signal timings are used to predict upstream phase duration [17].

For the coordinated phases at upstream intersection, phase durations can be obtained from the last optimization results. For the signal timings out of previous rolling horizon, the latest historical green time is assigned. The unknown green time duration for phase *p* in cycle *k* is then calculated as follows:

(21) $$\begin{array}{c} {\hat{g}}_{p,k}=g_{p,k-1},\;\forall p,k, \end{array}$$

where ${\hat{g}}_{p,k}$ is the estimated green time for upstream intersection and *g*_*p*,*k*−1_is the actual realized green time on cycle *k* − 1.

Based on the phase precedence constraints presented in Section 2.1, the estimated starting time of coordinated phase is obtained at upstream intersections. Therefore, the upstream platoon arrival time at current intersection can be calculated as follows:

(22) $$\begin{array}{c} R_{p,j,m}={\hat{t}}_{p,k}+t_{d},\;\forall \left(p,j,m\right)\in {\;}_{d},k, \end{array}$$

where ${\hat{t}}_{p,k}\;$is the estimated starting time for phase *p* at upstream intersection, *t*_*d*_  is the platoon travel time from upstream intersection to the current stop line, and Γ_*d*_ is the set of platoons which have not passed the upstream intersection. With the estimated arrival time of platoon discharged from upstream intersection as input, implicit coordination between neighboring intersections is fulfilled.

## 3. Simulation Case Study

To evaluate the effectiveness of the proposed model, a simulation and evaluation platform was developed in C++ language. The system contains VISSIM with COM (component object module) as a simulation module and IBM/CPLEX solver serving for optimization purpose. For every 30 seconds, vehicular information was extracted for the optimization, and CPLEX was used to find the optimal solution, so that the generated signal timings can be implemented into the VISSIM simulations [24].

### 3.1. Simulation Test Bed

**Figure 6:**
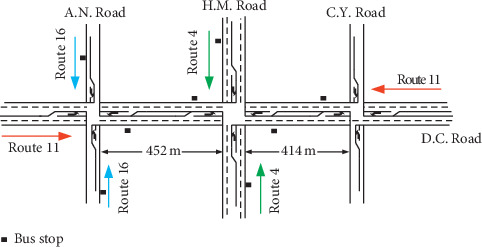
Network layout and bus routes.

Simulation tests were conducted based on the arterial segment of Dongchuan Road (D.C. Road), ranging from Cangyuan Road (C.Y. Road) to Anning Road (A.N. Road). As shown in Figure 6, three conflicting bus routes (routes 4, 11, and 16) are operated in the network, in which one route travels on the Dongchuan Road and the others travel on the cross streets. All far-side bus stops are located near the exit of upstream intersection. In the arterial network, D.C. Road/C.Y. Road and D.C. Road/A.N. Road are entry intersections of control zones, and vehicles arrive randomly at one coordinated phase (phase 2 or 6). In D.C. Road/H.M. Road, vehicles generally arrive in platoons at both coordinated phases subject to upstream signal controls [25].

Table 2 presents three demand scenarios to validate the proposed control model. The level of saturation is measured by Intersection Capacity Utilization (ICU) in SYNCHRO. It is assumed that all buses were equipped with CV technology, so that the information on bus location and speed is available. During the simulation tests, each bus sends priority requests before arriving at an intersection. The bus occupancy in this study changes from 20 to 30 and then to 40, under different demand scenarios, which may be based on the average number of passengers collected from APC (Automatic Passenger Counter) devices during the field implementation.

The background signal timing information was obtained from SYNCHRO, including cycle length, phase splits, and offsets. Fixed signal timings were served as the baseline for the proposed model. For each scenario, the simulation test runs with 10 different random seeds based on one-hour duration, including 10 min warm up period [26–28].

## 4. Results and Discussion

**Figure 7:**
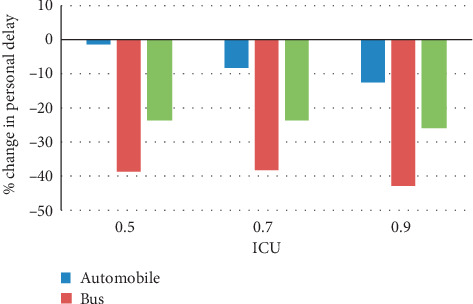
Percentage of change in person delay from SYNCHRO to person-based PASC under different traffic demands.

**Figure 8:**
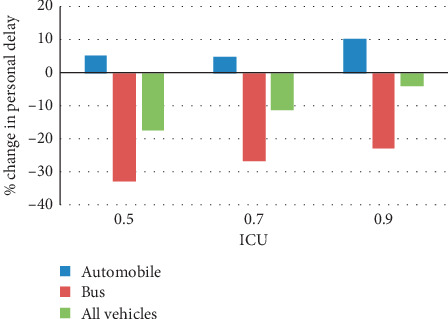
Percentage of change in person delay from vehicle-based to person-based PASC under different traffic demands.

Under the assumption of 100% penetration rate and average 40 bus passenger occupancy, the simulation test results with three different control strategies are listed in Table 3, namely, the fixed signal timings from SYNCHRO and the proposed control model with person-based and vehicle-based objective functions. Person-based objective function considers passenger occupancy in each bus, while vehicle-based one treats bus with the same weight with cars. Personal delay was chosen as the measurement performance of control strategy for automobiles, bus, and all vehicles. Figures 7 and 8 depict the percentage of change in personal delay from SYNCHRO to the proposed models, with findings summarized as follows.

As shown in Figure 7, the person-based PASC has obvious advantages compared with SYNCHRO, personal delay for each category of vehicle decreases with the rise of traffic demand. Specifically, under low traffic demand (ICU = 0.5), although the reduction of automobile passenger delay is not significant, the bus passengers experience nearly less 40% personal delay. More importantly, the decreasing trend of personal delay of all vehicles (more than 20%) shows that giving signal priority to bus passenger will not sacrifice the benefits of all automobile users. As a result, the person-based PASC actually reduces the delay of all the passengers.

Figure 8 presents the comparison between two PASC strategies with person-based and vehicle-based objective functions. First, the person-based PASC generated more personal delay for automobile passengers by 5%–10% compared with vehicle-based PASC, which is acceptable, since in transit priority, the automobile platoon may wait for longer time to give way to buses in other phases. With the increase of traffic demand, the disruption effect becomes more obvious. However, the person-based PASC still produce less personal delay for all vehicles in the network, again proving its ability to balance the passing priority of all users.

**Figure 9:**
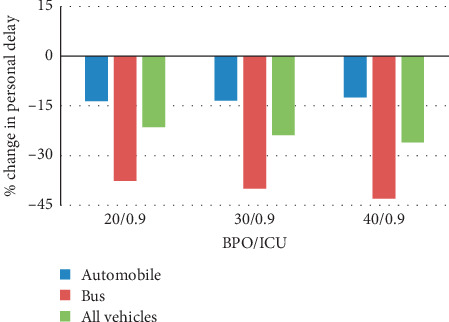
Percentage of change in person delay from SYNCHRO to person-based PASC with different BPOs.

To evaluate the impact of the fluctuation of bus passenger number to the controller's performance, three levels of the bus passenger occupancy (BPO), were set as 20, 30, and 40 passengers per bus, respectively. Within the heavy traffic demand (ICU = 0.9), the test results are presented in Figure 9. It can be figured out that the change of BPO has a limited impact on the optimization results. Personal delay of automobile passengers keeps almost unchanged, while the personal delay of bus passenger declines slightly with the increase of BPO. As a result, the overall road users would reduce personal delay by 20%.

**Figure 10:**
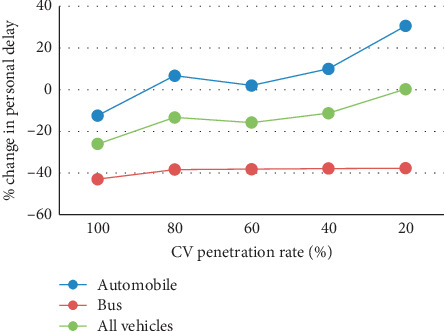
Percentage of change in person delay from SYNCHRO to person-based PASC with different CV penetration rates.

Since vehicle information collected by CV technology serves as an important input for platoon identification, the market penetration rate of CV devices largely influences the quality of signal timing. To test the model sensitivity to the penetration rate, the ideal assumption that all vehicles are equipped with CV devices was relaxed. Based on the penetration rate ranging from 20% to 100% with a 20% interval, simulation tests were conducted under high demand scenarios (ICU = 0.9; BPO = 40). Because all buses are generally equipped with GPS devices, only automobiles are assumed to subject to the penetration rate change.  Figure 10 illustrates the percentage of changes in person delay from SYNCHRO to person-based PASC under different penetration rates.

It can be figured out that personal delay of all types of vehicles increases as fewer vehicles are detected on the road. If the penetration rate is lower than 80%, PASC is likely to generate more personal delay to automobile passengers, and the increasing trend grows obviously under a lower penetration rate. This is probably caused by the estimation error in determining the position of the leading vehicle in the platoon, which can be solved by a more complicated platoon identification algorithm [13]. On the other hand, personal delay of the bus passenger rises slightly, which indicates that the estimation error of the front platoons does not influence the benefits of bus passengers. Considering PASC still reduces personal delay for passengers of all vehicles with more than 20% penetration rate, it is recommended that the obligatory CV penetration rate should be set at least as 20%.

## 5. Conclusions

This paper proposed an improved platoon-based adaptive control strategy to provide multimodal traffic management for signalized intersections, assuming that the connected vehicle information is available online. By introducing unified phase precedence constraints, PASC was not restricted by fixed cycle length and offset. A MILP optimization model was developed, in which platoon arrival and discharge dynamics were modeled for delay evaluation. With the communication between controllers, PASC provided implicit signal coordination of neighboring intersections for automobiles and buses.

A simulation and evaluation platform was developed to validate the proposed control strategy. The results indicated that person-based PASC successfully strikes a balance between automobile and bus passengers. At high demand scenario, person-based PASC reduces up to 40% bus passenger delay and 10% automobile passenger delay in comparison with SYNCHRO. Although the control performance of person-based PASC for automobile passengers is slightly inferior to vehicle-based PASC, it still reduces all personal delay by around 10%. Through sensitivity evaluation, it was found that the control performance keeps stable with the fluctuation of bus passenger number, and the minimal CV penetration rate is around 20%.

While the results are promising, limitations still exist within the proposed model, especially when the penetration rate is low. Therefore, how to improve the model robustness under a low penetration environment is a crucial question to be explored. Furthermore, additional progression mechanism and/or big data analytics should be incorporated into PASC to improve control performance in a large network [29, 30]. In future, PASC may be applied to a grid network to evaluate the effectiveness of implicit coordination.

## Figures and Tables

**Table 1:** Variables and data notations for the proposed optimization model.

Type	Symbol	Definition
Sets	*p* ∈ *P*	The set of phases
	*k* ∈ {1,2,…, *K*}	The set of cycles
	*j* ∈ *J*	The set of platoons
	*m* ∈ {*a*, *b*}	The set of traffic mode: a is the automobile, b is the bus
	(*p*, *j*, *m*) ∈ Γ	The set of *j*^th^ platoon at phase *p* and mode *m*
	Δ_1_, Δ_2_	The current phase in ring 1 and 2, respectively, Δ_1_, Δ_2_ ⊂ *P*
	Δ_0_	The set of past phases in cycle 1, Δ_0_ ⊂ *P*
	Δ_*p*_	The set of past phases in the current cycle

Decision variable	*t*_*p*,*k*_	Green starting time of phase *p* during cycle *k*
	*g*_*p*,*k*_	Green duration of phase *p* during cycle *k*
	*v*_*p*,*k*_	Phase duration time of phase *p* during cycle *k*
	*N*_*p*,*j*,*m*,*k*_^*r*^	Number of vehicles in residual platoon being cut from platoon (*p,j,m*) due to shortage of green time in cycle *k*(*N*_*p*,*j*,*m*,*k*_^*r*^ > 0)
	*x*_*p*,*j*,*m*,*k*_	Binary variable indicating whether platoon (*p*, *j*, *m*) is served before the end of phase *p* during cycle *k* (if *x*_*p*,*j*,*m*,*k*_=1, the platoon is served within cycle *k* else served in other cycles)
	*D*_*p*,*j*,*m*_^*l*^	Total delay by stopping the leading vehicle of platoon (*p*, *j*, *m*), *D*_*p*,*j*,*m*_^*l*^ > 0
	*D*_*p*,*j*,*m*_^*s*^	Total delay by splitting platoon (*p*, *j*, *m*), *D*_*p*,*j*,*m*_^*s*^ > 0
	*s*_*p*,*k*_	Slack time for phase *p* in cycle *k*

Data	*E*_*p*_	Elapsed green time for phase *p,p* ∈ Δ_0_
	*g*_*p*_^*N*^	Nominal green duration time for past phases in cycle 1
	*O*_*p*_	Initial starting time for phase *p*
	*Y*+*R*	Sum of yellow and red clearance time
	*G*_*p*_^min^, *G*_*p*_^max^	Minimum and maximum green time for phase *p*
	*R*_*p*,*j*,*m*_	Estimated arrival time at stop line for platoon (*p*, *j*, *m*)
	*N*_*p*,*j*,*m*_	Number of vehicles in platoon (*p*, *j*, *m*)
	*h*_*p*_	Saturated vehicle headway at stop line for phase *p*
	*M*	A large constant

**Table 2:** Demand scenario (veh/h).

Movement		ICU = 0.5			ICU = 0.7			ICU = 0.9		
		D.C./C.Y	D.C./H.M	D.C./A.N.	D.C./C.Y	D.C./H.M.	D.C./A.N.	D.C./C.Y	D.C./H.M.	D.C./A.N.
WB	L	52	131	50	69	225	122	154	200	190
	Th	431	502	515	835	852	659	1003	1008	1025
	R	88	42	113	123	122	86	128	79	109
EB	L	172	134	147	190	192	227	222	162	217
	Th	433	555	519	823	805	842	1138	1246	1362
	R	99	36	50	105	218	117	123	183	94
SB	L	134	192	59	196	206	78	170	350	64
	Th	93	259	123	154	447	163	163	542	163
	R	20	39	85	107	98	223	107	92	228
NB	L	68	90	128	96	144	227	116	190	234
	Th	66	190	165	154	402	106	156	510	139
	R	75	103	71	138	125	80	186	149	98

**Table 3:** Control performance under different methods (personal delay, seconds).

ICU	Vehicle type	SYNCHRO	PASC (person-based)	PASC (vehicle-based)
0.5	Automobile	24.2	23.9	22.7
	Bus	34.7	21.3	31.6
	All vehicles	29.8	22.6	27.4
0.7	Automobile	28.5	26.2	25.0
	Bus	40.6	25.0	34.1
	All vehicles	33.7	25.7	29.0
0.9	Automobile	35.5	31.1	28.2
	Bus	51.8	29.6	38.3
	All vehicles	41.3	305	31.8

## Data Availability

The data used to support the findings of the study are available from the corresponding author upon request.
